# Temporal Trends in the Use of Biological Agents in Patients with Inflammatory Bowel Disease: Real-World Data from a Tertiary Inflammatory Bowel Disease Greek Center During a 5-Year Period

**DOI:** 10.3390/jcm14041357

**Published:** 2025-02-18

**Authors:** Panagiotis Markopoulos, Aikaterini Gaki, Georgios Kokkotis, Konstantina Chalakatevaki, Nikolaos Kioulos, Vasso Kitsou, Constantinos Tsitsigiannis, Michael Gizis, Paraskevi Prapa, Stamatina-Lydia Chatzinikolaou, Efrosini Laoudi, Ioannis Koutsounas, Giorgos Bamias

**Affiliations:** 1 GI Unit, 3rd Academic Department of Internal Medicine, National and Kapodistrian University of Athens, Sotiria Hospital, 15772 Athens, Greece; panosmarkmd@gmail.com (P.M.); katgaki@gmail.com (A.G.); gkokkot@gmail.com (G.K.); kchalakatevaki@gmail.com (K.C.); nikolaoskioulos@hotmail.gr (N.K.); vassosgkp@gmail.com (V.K.); kostastsitsi@gmail.com (C.T.); gizism@gmail.com (M.G.); eprapa@hotmail.com (P.P.); matinahatzinicolaou@gmail.com (S.-L.C.); laoudif@gmail.com (E.L.); john_koutsounas@yahoo.gr (I.K.); 2 Gastroenterology Department, “Metaxa” Memorial Hospital, 18537 Piraeus, Greece

**Keywords:** inflammatory bowel disease, biologicals, treatment persistence, dose intensification, time trends

## Abstract

**Background/Objectives:** Therapeutic management of inflammatory bowel diseases (IBD) is rapidly evolving in the era of novel biological therapies. However, real-world data relating to the usage trends and treatment persistence remain inconsistent. This study aimed to investigate trends in biological use, dose intensification, and treatment persistence in IBD patients, who received treatment in a large tertiary center in Greece. **Methods:** Patients with IBD who underwent at least one biological treatment between 2018 and 2022 were included in this retrospective study. Data on patients’ demographics, type of disease, use of biologicals, dose intensification, and treatment persistence were analyzed for time trends. **Results:** Data from 409 patients with IBD (mean age 39 (range 17–87), female 51%, 56.9% CD, mean duration of disease: 9.3 years) were included in the study. The number of patients on biologics was raised from 133 in 2018 to 368 in 2022 (a 28.1% yearly increase), while the percentage of patients who were treated with anti-TNF biosimilars increased to >60% of the total anti-TNF population in 2022. We observed a gradual increase in non-anti-TNF therapies in bio-naïve patients, in particular vedolizumab (46% of all biologicals in UC; 16% in CD) and ustekinumab (16.3% of all biologicals in UC, 31% in CD). The 3-year persistence rate of IFX was 64% in CD and 56% in UC, whereas it was 61% for ADA in CD. Dose intensification of anti-TNF was efficient in >50% of CD patients and >30% of UC patients; however, the majority of patients who required dose escalation within the first year eventually became unresponsive. The 3-year persistence of vedolizumab as a first-line treatment was 82% for CD and 69% for UC, respectively. The 3-year persistence of ustekinumab as first-line treatment for CD was 65%. No significant differences regarding the efficacy of anti-TNF, ustekinumab, or vedolizumab were detected when they were used as first-line treatments for Crohn’s disease; similarly, no significant differences were detected between infliximab and vedolizumab as first-line treatments for UC. **Conclusions:** There was a gradual increase in the use of biologicals, including biosimilars, between the years 2018–2022, reflecting adherence to current guidance with adoption of an early escalation strategy. Newer, post-anti-TNF biologics such as vedolizumab and ustekinumab have been rapidly incorporated into therapeutic approaches for both CD and UC.

## 1. Introduction

Inflammatory bowel diseases (IBDs), namely Crohn’s disease (CD) and ulcerative colitis (UC), are chronic inflammatory disorders which mainly affect the gastrointestinal tract, and likely originate from an abnormal inflammatory process against antigens of the normal intestinal flora, with environmental and genetic factors playing a pivotal role [1]. Since 1990s, when the anti-TNF agents (infliximab, adalimumab) were approved for the treatment of IBD, the armamentarium has been expanded with novel biologic therapies and molecules, including the anti-interleukin-12/23 ustekinumab and the gut-selective anti-α4β7-integrin vedolizumab [2].

Clinical guidelines have rapidly incorporated novel therapies and provided treatment recommendations with regard to their use as both first- and later-line therapies for Crohn’s disease (CD) and ulcerative colitis (UC) [3,4,5].

The evolution of biologics, along with the supporting evidence from guidelines that position them primarily within the early stages of the disease, has significantly improved patients’ outcomes and quality of life [6]. Nevertheless, a substantial number of patients will have to cease their medication, either due to suboptimal response, or due to adverse events [2]. Therefore, it is important for the physician to carefully select the first line of biologics and determine which therapy to add later, as this decision may impact the persistence of the patient’s response to their medication [2,7].

However, positioning biologics in the IBD therapy may be challenging due to the limited data on the comparative effectiveness of biologics, from high-quality head-to-head studies, which remain the most valid approach in this context [8,9]. Real-world retrospective studies may provide significant evidence and partially fill this gap by comparing the persistence of medications in patient groups when they are used either as first- or later-line therapies [2].

In this study, we aimed (a) to record the temporal trends in the prescription of biologics in our center between the years 2018 and 2022, (b) to depict our practice in positioning the biologics and maintaining them via dose intensifications, when necessary, (c) to record the 3-year persistence of each medication, given that the mean duration of follow-up was 3.8 years, and (d) to compare the effectiveness of different biologics in bio-naïve and bio-experienced patients.

## 2. Materials and Methods

### 2.1. Population

This was a retrospective, observational cohort study carried out at a single tertiary IBD center in Athens, Greece. All the participants had a definitive diagnosis of IBD (CD or UC) and were treated with at least one biologic between the years 2018–2022. The diagnosis of IBD was based on the criteria established by ECCO [10]. The Montreal classification was adopted for disease phenotyping [11].

The patients were regularly followed up with laboratory tests, endoscopy, and/or imaging, when necessary, and non-responders were switched to the next line of treatment as per the standard care practice. Data on patients’ demographics, disease phenotype, type of treatment, dose and intervals of administration, dose intensification, treatment persistence, and clinical outcomes including surgery were retrieved from the local database and the time trends were analyzed. The mean duration of follow-up was 3.8 years (a range of 1–6 years)

### 2.2. Statistical Analysis

All calculations were performed using the SPSS 21.0 software package (SPSS Inc., Chicago, IL, USA). Percentages were computed for discrete data and mean values ± standard deviation (SD) or medians with interquartile ranges (IQRs) were calculated for continuous data. Differences between independent groups were traced with the use of chi2 for categorical variables, Student’s *t*-test for normally distributed values and the Mann–Whitney U test for non-normally distributed values. A one-way ANOVA was used to compare the means of multiple groups. Kaplan–Meier plots were used to depict the survival-free treatment discontinuation of the biologic treatments when they were used as first or second line of therapy for both diseases. The differences in distributions between medications were compared using the Log-rank test. A value of *p* < 0.05 was considered statistically significant.

## 3. Results

Data from 409 consecutive patients were included in the study. Of those, 244 were diagnosed with CD (59.6%), 51% were female, and the mean age was 39 years. Patients’ baseline characteristics are shown in Table 1.

### 3.1. Temporal Trends

**Figure 1:**
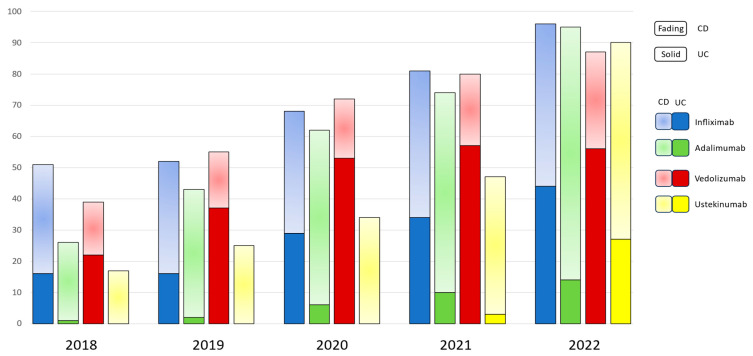
Overall trend of prescribed biologics in patients with IBD from 2018 through 2022.

There was a cumulative annual growth rate of 28.1% in the number of patients with IBD who were receiving biologics, increasing from 133 individuals in 2018 (55% of the Department’s IBD population) to 368 individuals in 2022 (78% of the Department’s IBD population) (Figure 1). Notably, no subcutaneous forms of infliximab or vedolizumab were available in Greece; thus, all patients on these medications received the IV form exclusively. Anti-TNF agents (namely infliximab and adalimumab) retained their central position among advanced therapies in CD, as more than 50% of the CD patients were treated with either infliximab or adalimumab, each year. Adalimumab was medication most frequently prescribed to treat CD, presenting continuous prominence in the years 2019 (34.2% of patients), 2020 (37.8% of patients), 2021 (35.9% of patients), and 2022 (35.7% of patients). There was also a gradual increase in the trend of ustekinumab administration, which eventually became the second most prescribed medication amongst CD patients, with the 18% of patients prescribed this medication in 2018 increasing to 27.8% of patients in 2022 (Figure 1).

**Figure 2:**
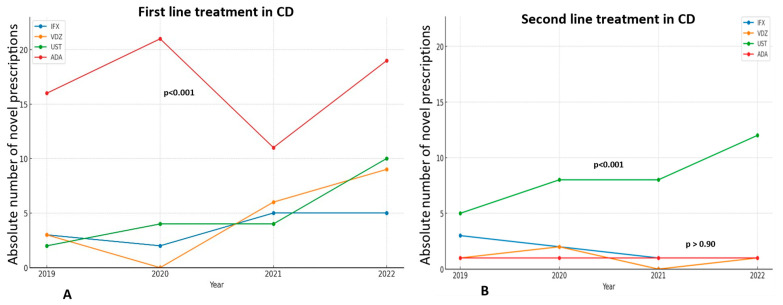
The trend in novel prescriptions of each biologic as a first treatment line (**A**) or as a second treatment line (**B**) in CD, through the years 2019–2022. ADA and UST are significantly the most prescribed medications as first and second treatment lines, respectively.

Among bio-naïve patients with CD, adalimumab was significantly preferred to other medications (*p* = 0.00) (Figure 2A), while ustekinumab was the biologic most frequently prescribed for bio-experienced CD patients (*p* < 0.001) (Figure 2B).

**Figure 3:**
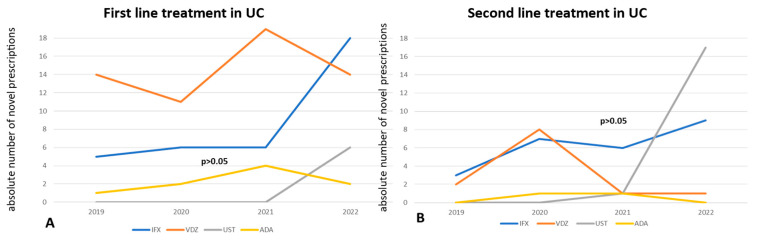
The trend in novel prescriptions of each biologic as a first treatment line (**A**) or as a second treatment line (**B**) in UC, through the years 2019–2022. VDZ is significantly the most prescribed medication as first treatment line, while UST is numerically the most frequently prescribed second-line treatment.

In UC, vedolizumab is the most frequently prescribed medication overall, particularly in 2018 (56.4% of patients), in 2019 (67.2%), and 2020 (60.2%), although there was a slight decline in its prescription trend in the subsequent years 2021 and 2022 (Figure 1). More specifically, in bio-naïve UC patients, vedolizumab maintains its central position as the first-line treatment, despite fluctuations in its prescription trend (*p* < 0.001), while infliximab showed a sharp increase in prescriptions in 2022, after a continuous plateau in the previous years (Figure 3A). On the other hand, there has been no significant difference in the trends of biologics use among bio-experienced patients, although the use of ustekinumab sharply increased in 2022, making it the most prescribed medication from a numerical standpoint (Figure 3B).

### 3.2. Biosimilars

There was a gradual increase in the prescription of anti-TNF biosimilars over the years, from 33% (17 of 51 IBD patients on anti-TNF) in 2018 to 67% (61 of 91 IBD patients on anti-TNF) in 2022. More specifically, among patients on IFX, 33% (17/51) were treated with a biosimilar in 2018, 48% (25/52) in 2019, 45% (31/68) in 2020, 39% (32/81) in 2021 and 67% (61/91) in 2022. Similarly, among patients on ADA, 4% (1/26) were treated with a biosimilar in 2018, 2% (1/43) in 2019, 17% (11/62) in 2020, 27% (17/62) in 2021, and 47% (45/95) in 2022. No biosimilars of vedolizumab and ustekinumab were available in Greece during that period (Detailed data on biosimilars administration on Supplementary Materials).

### 3.3. Dose Intensification

Dose intensification was defined as any regimen differing from the standard 5 mg/kg every 8 weeks for infliximab or 40 mg fortnightly for adalimumab or 300 mg every 8 weeks for vedolizumab, or 90 mg every 8 weeks for ustekinumab. Dose intensification was applied to patients on anti-TNF therapy and active intestinal disease and/or arthropathy, either empirically or guided by subtherapeutic trough levels. Overall, 21 out of 58 (36.2%) CD patients on IFX and 34 of 95 (35.7%) CD patients on ADA underwent dose intensifications, at some point, between 2018 and 2022. The mean duration of treatment before escalation was 20.4 months (9–60 months) for CD patients on IFX and 18.52 (0–63 months) months for CD patients on ADA. (In UC, 22 of 63 (34.9%) patients on IFX and 8 of 15 (53.3%) patients on ADA underwent dose optimization, with the mean duration of treatment before escalation being 11.55 months (0–42 months) for IFX and 19.87 months (3–46 months) for ADA. Additionally, 13 out of 21 (62%) CD patients on IFX and 18 out of 34 (52%) CD patients on ADA recaptured their response after dose optimization. On the other hand, only 7 out of 21 (31%) UC patients on IFX and 4 out of 8 (50%) UC patients on ADA maintained their treatment post-escalation. Early escalation (within 12 months after treatment initiation) negatively affected therapy persistence, as these patients were significantly more likely to become less responsive compared to those with late escalation (>12 months after treatment initiation) (*p* < 0.001) (Table 2).

The number of patients on vedolizumab and ustekinumab that underwent dose escalation was much smaller (6% of CD patients and 10% of UC patients on vedolizumab and 10% of CD patients and 8% of UC patients on ustekinumab, respectively).

### 3.4. Persistence

Persistence was defined by the proportion of patients who remained on the index treatment without a gap of >180 days [12]. The 3-year persistence of infliximab was 64% in Crohn’s disease patients and 56% in patients with ulcerative colitis, while that of adalimumab was 61% in Crohn’s disease patients. The 3-year persistence of vedolizumab as a first-line treatment was 82% for Crohn’s disease and 69% for ulcerative colitis, respectively. The 3-year persistence of ustekinumab as first-line treatment in Crohn’s disease was 65%; no data on the long-term persistence are currently available for UC, due to the delayed approval of the medication in Greece. However, the 2-year persistence of the medication stood at 75%.

### 3.5. Survival Analysis

**Figure 4:**
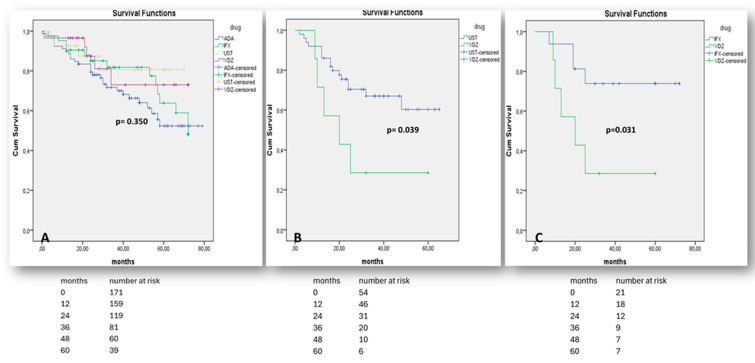
“Kaplan–Meier” curves demonstrate survival free of treatment discontinuation in CD patients, when biologics are prescribed as the 1st treatment line (**A**), as the 2nd treatment line after non-response to any anti-TNF agent (**B**), or after non-response to adalimumab only (**C**). “Log-Rank” *p* < 0.05 is considered as statistically significant.

We used discontinuation-free survival analysis to compare the effectiveness of biologics when administered as first-line or later-line treatments in Crohn’s disease and Ulcerative colitis patients. All comparisons between biologics were performed in patient groups with no significant heterogeneity in terms of sex, mean age, duration of disease, and smoking history, using the chi-square test (*p* > 0.05). In CD, the groups consisted of patients with isolated luminal disease and no evidence of perianal/fistulizing phenotype and/or concomitant extraintestinal manifestations. Interestingly, no significant difference in the effectiveness of therapy with any kind of biologics (infliximab, adalimumab, vedolizumab, and ustekinumab) was observed when used in bio-naïve patients with luminal Crohn’s disease (*p* = 0.350—Figure 4A). In anti-TNF experienced patients with CD, ustekinumab was significantly superior to vedolizumab (*p* = 0.039—Figure 4B), while even infliximab was more effective than vedolizumab in patients with prior failure to respond to adalimumab (*p* = 0.031—Figure 4C).

**Figure 5:**
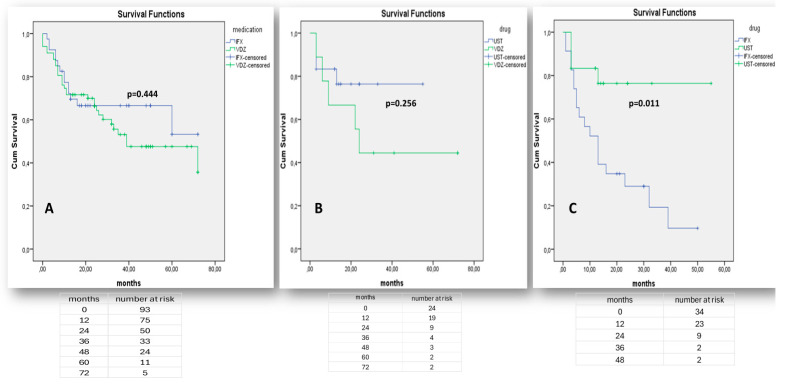
“Kaplan–Meier” curves demonstrate survival free of treatment discontinuation in UC patients, when biologics (IFX, VDZ) are prescribed as the 1st treatment line (**A**), when biologics (UST, VDZ) are prescribed as the 2nd treatment line after non-response to any anti-TNF agent (**B**), or when biologics (IFX, UST) are prescribed as the 2nd line treatment after non-response to vedolizumab (**C**). “Log-Rank” *p* < 0.05 is considered as statistically significant.

One the other hand, there was no significant difference in the effectiveness of either vedolizumab or infliximab when they were used as first-line medications in moderate-to-severe UC (*p* = 0.444—Figure 5A). As the number of bio-naïve patients on adalimumab was small and could not be adjusted for confounding factors, including active arthropathy, the efficacy of adalimumab in UC in comparison to other therapies was not assessed. Both vedolizumab and ustekinumab were similarly effective when administered after anti-TNF failure (*p* = 0.256, Figure 5B) and ustekinumab was significantly superior to infliximab in terms of prior non-response to vedolizumab (*p* = 0.011, Figure 5C).

## 4. Discussion

In this retrospective study, we have presented data on the use and clinical outcomes of biologic therapies in 409 patients over the years 2018–2022. At this time, there was a cumulative increase in the use of biologics by almost 29% yearly, which launched an increase in the number of patients, which rose from 133 individuals in 2018 to 368 in 2022. There was also a cumulative increase in the use of biologics other than anti-TNF in bio-naïve patients, with vedolizumab and ustekinumab being the preferred medications in 40.9% UC patients and 25% CD patients, respectively.

The increasing use of biologics clearly reflects the increasing incidence of inflammatory bowel disease in various Western countries, including Greece, over the past few years [13,14]. More specifically, an ECCO-EpiCom study including data from a specific region in Greece demonstrated a clear increase in the incidence of both diseases, which increased by 0.4–0.6/100,000 inhabitants over the past decade [15].

Moreover, at that time, there was increasing evidence to support a “top-down” rather than a “step-up” process in the management of moderate-to-severe cases of inflammatory bowel diseases, which was subsequently adopted by the clinical guidelines [3,4,5,16]. Hence, the standard of care includes the early administration of biologics, particularly in patients presenting with increased inflammatory burden. The increasing use of biologics from the year 2018 to 2022 reflects our department’s gradual adherence to the “top-down” approach as a standard practice of care.

Undoubtedly, the licensing of cheaper biosimilar anti-TNF agents and the massive amount of reported evidence of their equality to the original molecules in terms of efficacy and safety have supported the approach of early escalation. Our study highlights a gradual incorporation of biosimilars in our clinical practice to retain the lion’s share of doses (67% in 2022) and minimize the cost of services for the National Healthcare System. Understandably, the exploitation of biosimilars was gradual (from 33% in 2018 to 67% in 2022), as the evidence regarding the use and the feasibility of switching from the original to the biosimilar treatment became more supportive [17,18,19].

In this study, we also recorded data on dose intensification of biologics. Dose optimization of anti-TNF agents was guided by either the assessment of anti-TNF trough and antibody levels or empirically in patients with active IBD and/or active arthropathy. Dose optimization of vedolizumab and ustekinumab was guided empirically in patients who exhibited at least a partial response to the induction scheme. Our findings demonstrated that dose intensification may increase the response rate of the patients and maintain therapy in >50% of patients with CD and in at least >30% of patients with UC. These findings align with the extensively published data regarding the value of the anti-TNF dose intensification in the management of patients with IBD that has been published over the past decade [20,21,22]. However, in our study, most patients treated with adalimumab and almost all the patients taking infliximab, who experienced dose escalation within the first year, finally became non-responsive during the long-term follow up, even if they had temporarily presented clinical improvement. Early escalation (within the first 12 months) was proved as a definitive risk factor for treatment discontinuation, regardless of whether dose intensification was guided by active IBD or arthropathy. This contradicts the findings of several metanalyses, in which the authors have speculated on the efficacy of dose intensification within the first 6 months, albeit they focused on secondary non-responders [22,23]. The cornerstone of efficient dose escalation is particularly relevant in cases of secondary loss of response, especially when it is attributed to suboptimal trough levels [24]. Active disease, despite optimal trough levels, may reflect the presence of inflammatory pathways, other than TNF-mediated ones, thus requiring medications outside of the anti-TNF class [24]. Potential explanations for our findings include the increased inflammatory burden in most patients requiring early escalation, which is an established risk factor for treatment failure, alongside occasional misleading evidence of primary response to treatment, based on subjective criteria, such as the patients’ clinical improvement.

We also studied the long-term persistence of biologic medications as a measure for long-term efficacy when used as either a first or second line of treatment. Although this kind of study is not comparable to high-quality head-to-head trials, such studies may provide data from real world situations that are not adequately represented in clinical trials. Data from the SEAVUE head-to-head trial highlights the non-inferiority of ustekinumab compared to the standard dose of adalimumab as a first-line treatment for Crohn’s disease [9]. In our study, there was no statistically significant difference in the persistence of any of the medications studied (infliximab, adalimumab, vedolizumab, and ustekinumab) when they were used as first-line treatments for Crohn’s disease. However, no adjustment for cofactors related to the disease phenotype could be made, as anti-TNF was exclusively used as the first-line treatment for all the patients with perianal involvement and/or arthritic manifestations. Therefore, the above findings are limited to patients with luminal disease (L1–L4), after adjusting for cofactors related to the patients’ population (age, sex, smoking history) and disease extent and duration. These findings align with those from similar cohort studies [25,26], but also from a large UK cohort study which included more than 13,000 patients [27] and demonstrated no statistical significance in the effectiveness of biologics when they were used as first-line treatments for luminal disease.

Moreover, our study demonstrated no significant difference in the long-term persistence of infliximab and vedolizumab when they were used as first-line treatments for UC (*p* = 0.444). These results contradict growing evidence from the VARSITY head-to-head clinical trial, as well as several real-world studies and meta-analyses, including the UK cohort study, which promote vedolizumab as more efficacious than anti-TNF agents, particularly adalimumab, in UC [8,28,29]. Nevertheless, our findings are supported by several network meta-analyses of randomized trials, which did not demonstrate differences in efficacy between first-line VDZ and IFX in UC [26,30]. In addition, Peyrin-Biroulet et al. underlined the similar efficacy of subcutaneous infliximab and vedolizumab as first-line treatments in moderate-to-severe UC [31]. As our clinical practice has been aligned with the current evidence, our cohort includes only 12 UC patients on adalimumab, who had achieved deep remission on 5-ASA, before initiating anti-TNF to treat extraintestinal manifestations. Given this confounding factor, adalimumab was not compared to other biologics.

Our study also included real-world data on comparative effectiveness of drug sequencing. With regard to CD, there was a statistically significant difference in the long-term persistence of ustekinumab compared to vedolizumab in patients with prior anti-TNF exposure (*p* = 0.039). Vedolizumab was also significantly inferior to infliximab (*p* = 0.031), when used as second-line treatment in patients who had previously failed to respond to adalimumab. Moreover, there was no significant difference in the persistence of ustekinumab and infliximab when they were administered as second-line treatments in patients with previous experience taking adalimumab. Several cohort studies have provided conflicting data on the comparative effectiveness of vedolizumab and ustekinumab as second-line treatments for CD. Large cohort studies have failed to show any significant difference between these two agents, although other studies have demonstrated the clear superiority of ustekinumab over vedolizumab in anti-TNF non-responders [27,32,33].

In terms of UC, our data suggest that vedolizumab and ustekinumab are similarly effective as second-line treatments post anti-TNF failure (*p* = 0.382); our data also suggest that ustekinumab is more effective than infliximab in patients with previous failure to respond to vedolizumab (*p* = 0.008). Several real-world studies have stipulated on either the lack of superiority between biologics or on ustekinumab’s superiority after anti-TNF failure [30,34,35,36]. However, to the best of our knowledge, aside from an abstract reference in the ECCO congress in 2024 [37], this is the first study to compare ustekinumab and infliximab following vedolizumab failure in UC. This study also highlights the significant superiority of ustekinumab over infliximab over a two-year follow-up period, despite the relatively small sample size. Undoubtedly, further prospective studies with larger numbers of patients are needed to validate these results.

The study’s limitations include the risk of recall bias inherent in retrospective studies, alongside the lack of data from other IBD centers, which have limited the number of patients in specific sub-groups. In addition, although we have confidently eliminated covariates such as the concomitant use of steroids during follow-up, or delays in drug cessation due to the varying thresholds for switching implemented by different physicians, missing data can occur, especially for the years 2020–2022, wherein restrictions from the COVID pandemic may have complicated patients’ regular follow-up, thus affecting the standard of care [38]. Therefore, additional covariates might have affected treatment outcomes that we have not yet identified.

## 5. Conclusions

This is the first Greek study which provided data on temporal trends, dose intensification, and the comparative effectiveness of biologics used both as first and second lines of treatment. There was a gradual increase in the prescription of biologics in patients with IBD between 2018 and 2022. Anti-TNFs have retained their central position in the armamentarium, although novel therapies including vedolizumab and ustekinumab have been used with increasing frequency in both bio-naïve and bio-experienced patients. The persistence of biologics as first-line treatments did not differ significantly in Crohn’s disease and ulcerative colitis patients, when selected carefully. Ustekinumab has proved significantly superior as a second-line treatment compared to vedolizumab and to infliximab in CD and UC, respectively. Further prospective studies are essential, as these will allow us to establish a valid strategy regarding the positioning of biologics in IBD.

## Figures and Tables

**Table 1:** Demographic and clinical characteristics of inflammatory bowel disease patients included in the study. ADA: adalimumab, IFX: infliximab, VDZ: vedolizumab, UST: ustekinumab, CD: Crohn’s disease, and UC: ulcerative colitis.

Mean age (years)	39 (17–87)
Females (%)	210/409 (51)
CD/UC (%)	244/165 (59.6/40.4)
Mean disease duration (years)	9.3
Smokers (%)	142 (34.7)
**Disease location** (%)	
E1/E2/E3	17/61/87 (10.3/36.9/52.7)
L1	126 (51.6)
L2	15 (6.1)
L3	103 (42.2)
L4	41 (16.8)
**Disease behavior** (%)	
B1	173 (70.9)
B2	57 (23.2)
B3	14 (5.7)
Perianal involvement (*p*)	26 (10.6)
**Extraintestinal manifestations** (%)	133 (32.5)
Arthritic	95 (38.9)
Skin	31 (12.7)
Ocular	18 (7.3)
**Major IBD-related surgery**	53 (12.9)
**Biologic Treatment (1st line)** (%) **CD**	
ADA	96 (39.3)
IFX	61 (27.4)
VDZ	30 (12.2)
UST	57 (23.3)
**Biologic Treatment (1st line)** (%) **UC**	
ADA	15 (9)
IFX	67 (40.6)
VDZ	72 (43.6)
UST	11 (6.6)
**Biologic Treatment (2nd line)** (%) **CD**	
ADA	2 (2.6)
IFX	16 (21.3)
VDZ	7 (9.3)
UST	50 (66.6)
**Biologic Treatment (2nd line)** (%) **UC**	
ADA	4 (7)
IFX	24 (42.1)
VDZ	9(15.7)
UST	20 (35)

**Table 2:** Patients with IBD on dose intensification of their anti-TNF therapy (both infliximab and adalimumab). UC: ulcerative colitis, CD: Crohn’s disease, IFX: infliximab, ADA: adalimumab. Escalation > 12 months: when the dose was intensified ≥ 12 months after the initiation of anti-TNF therapy. Escalation < 12 months: when the dose was intensified < 12 months after the initiation of anti-TNF therapy. Chi^2^ test was performed to explore significant differences between the groups with escalation > 12 months and escalation < 12 months for each anti-TNF agent. *p* < 0.05 is considered statistically significant.

Type of Disease	Number of Patients Undergoing Escalation	
	IFX	ADA
UC	22/63 (34.9%)	8/15 (53.3%)
Mean duration before escalation (months)	11.55 (0–42)	19.87 (3–46)
Escalation ≥ 12 months	10/22 (45%)	6/8 (75%)
	LOSS OF RESPONSE	LOSS OF RESPONSE
	4/10 (40%)	2/6 (33.3%)
Escalation < 12 months	12/22 (55%)	2/8 (25%)
	LOSS OF RESPONSE	LOSS OF RESPONSE
	11/12 (91.67%), *p* < 0.001	2/2 (100%), *p* < 0.001
CD	21/58 (36.2%)	34/95 (35.7%)
Mean duration before escalation (months)	20.4 (9–60)	18.52 (0–63)
Escalation ≥ 12 months	16/21 (76.19%)	21/34 (61.7%)
	LOSS OF RESPONSE	LOSS OF RESPONSE
	3/16 (18.7%)	8/21 (38%)
Escalation < 12 months	5/21 (23.8%)	13/34 (38.2%)
	LOSS OF RESPONSE	LOSS OF RESPONSE
	5/5 (100%), *p* < 0.001	8/13 (61.5%), *p* = 0.02

## Data Availability

The data underlying this article will be shared on reasonable request to the corresponding author.

## Supplementary Materials

The following supporting information can be downloaded at https://www.mdpi.com/article/10.3390/jcm14041357/s1.
