# Supplementary material for: Temporal Trends in the Use of Biological Agents in Patients with Inflammatory Bowel Disease: Real-World Data from a Tertiary Inflammatory Bowel Disease Greek Center During a 5-Year Period
Source: J Clin Med. 2025 Feb 18;14(4):1357. doi: 10.3390/jcm14041357 (PMC11856159; doi:10.3390/jcm14041357)
Supplement: Supplementary file 1 [file jcm-14-01357-s001.zip › jcm-3405408-supplementary.pdf]

number of patients on biologics from 2018 to 2022					
	year				
**biologic**	**2018**	**2019**	**2020**	**2021**	**2022**
**IFX**	51 (38.3%)	52 (29.7%)	68 (28.8)	81 (28.7%)	96 (26%)
**ADA**	26 (19.5%)	43 (24.5%)	62 (26.3%)	74 (26.2%)	95 (25.8%)
**VDZ**	39 (29.3%)	55 (31.4%)	72 (30.5%)	80 (28.3%)	87 23.6%)
**UST**	17 (12.8%)	25 (14.3%)	34 (14.4%)	47 (16.6%)	90 (24.4%)
**total**	133	175	236	282	368

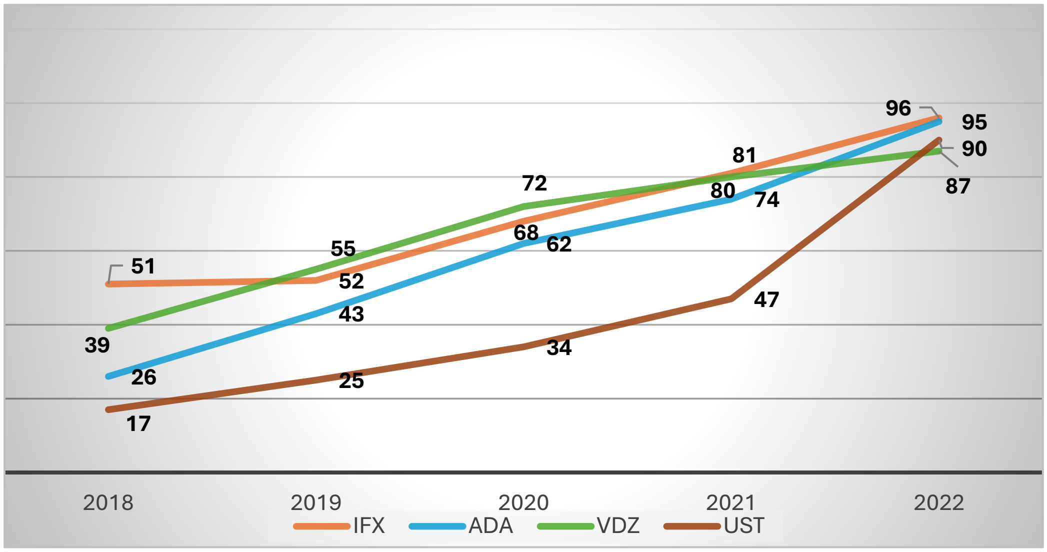

Figure S1: The overall trend of biologics prescribed in all patients with IBD from 2018 to 2022

IFX: infliximab, ADA: adalimumab, VDZ: vedolizumab, UST: ustekinumab

number of patients with UC on biologics between 2018 -2022					
	Year				
**biologic**	**2018**	**2019**	**2020**	**2021**	**2022**
**IFX**	16 (41%)	16 (29%)	29 (32.9%)	34 (32.6%)	44 (31.2%)
**ADA**	1 (0.02%)	2 (0.03%)	6 (0.06%)	10 (0.09%)	14 (0.09%)
**VDZ**	22 (56.4%)	37 (67.2%)	53 (60.2%)	57 (54.8%)	56 (39.7%)
**UST**	0 (0%)	0 (0%)	0 (0%)	3 (0.02%)	27 (19.1%)
**total**	39	55	88	104	141

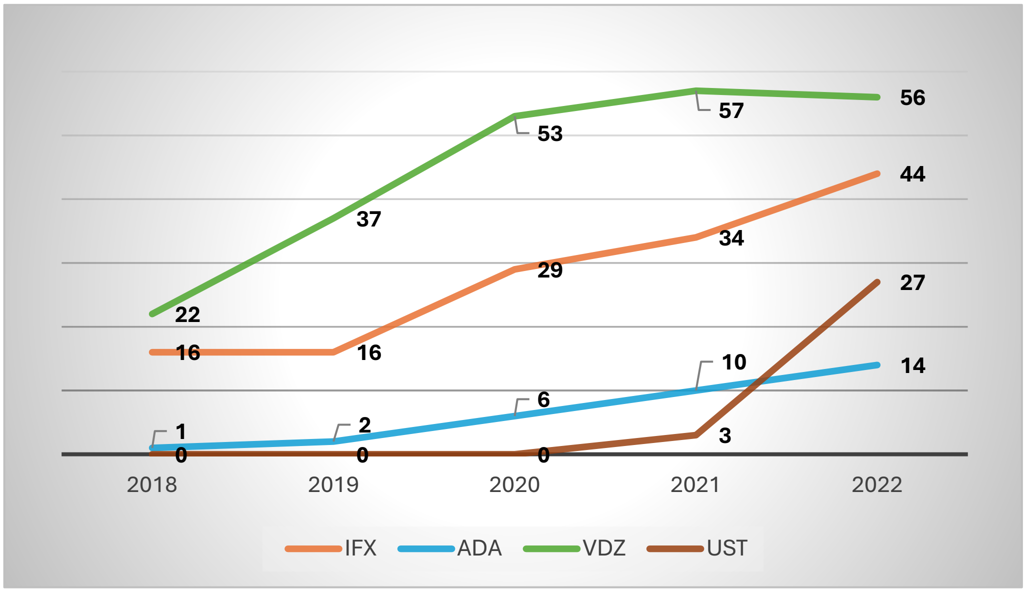

Figure S2: total number of UC patients on each biologic from 2018 to 2022.

IFX: infliximab, ADA: adalimumab, VDZ: vedolizumab, UST: ustekinumab

number of patients with CD on biologics from 2018 to 2022					
	Year				
biologic	2018	2019	2020	2021	2022
IFX	35 (37.2%)	36 (30%)	39 (26.3%)	47 (26.4%)	52 (22.9%)
ADA	25 (26.5%)	41 (34.2%)	56 (37.8%)	64 (35.9%)	81 (35.7%)
VDZ	17 (18%)	18 (15%)	19 (12.8%)	23 (12.9%)	31 (13.6%)
UST	17 (18%)	25 (20%)	34 (22.9%)	44 (24.7%)	63 (27.8%)
total	94	120	148	178	227

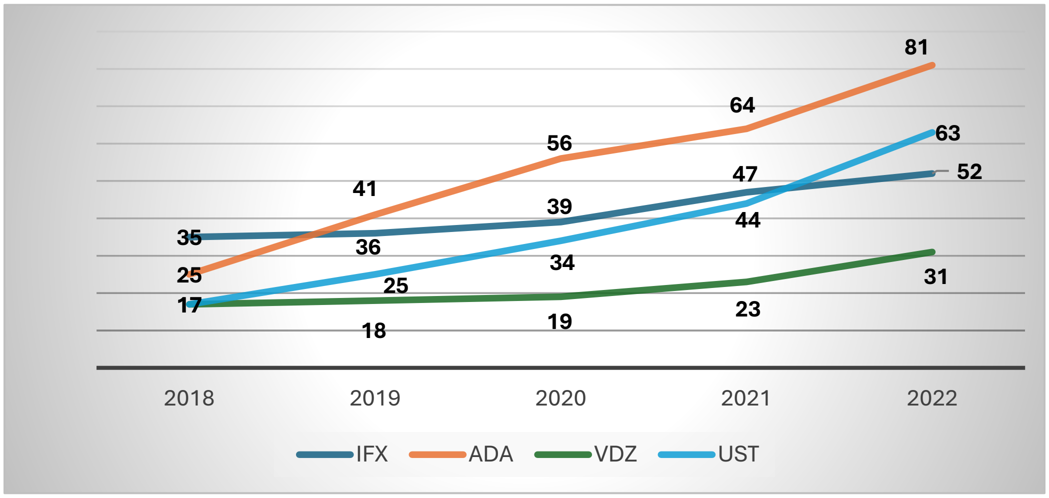

Figure S3: total number of CD patients on each biologic from 2018 to 2022.

IFX: infliximab, ADA: adalimumab, VDZ: vedolizumab, UST: ustekinumab

YEAR	NOVEL PRESCRIPTION OF BIOLOGICS FROM 2019-2022												
	IFX			VDZ			UST			ADA			
	treatment line			treatment line			treatment line			treatment line			
	1st	2nd	3rd	1st	2nd	3rd	1st	2nd	3rd	1st	2nd	3rd	Total
2019	3	3	1	3	1	0	2	5	1	16	1	0	**36**
2020	2	2	1	0	2	0	4	8	2	21	1	0	**43**
2021	5	1	3	6	0	2	4	8	2	11	1	0	**43**
2022	5	1	2	9	1	0	10	12	3	19	1	0	**63**

Table S1: Trends in the novel prescription of each biologic in both bio-naïve and bio-experienced CD patients in years 2019-2022. ADA remains the preferred medication for bio-naïve patients, while UST is the most prescribed biologic as a 2nd line of treatment.

IFX: infliximab, ADA: adalimumab, VDZ: vedolizumab, UST: ustekinumab

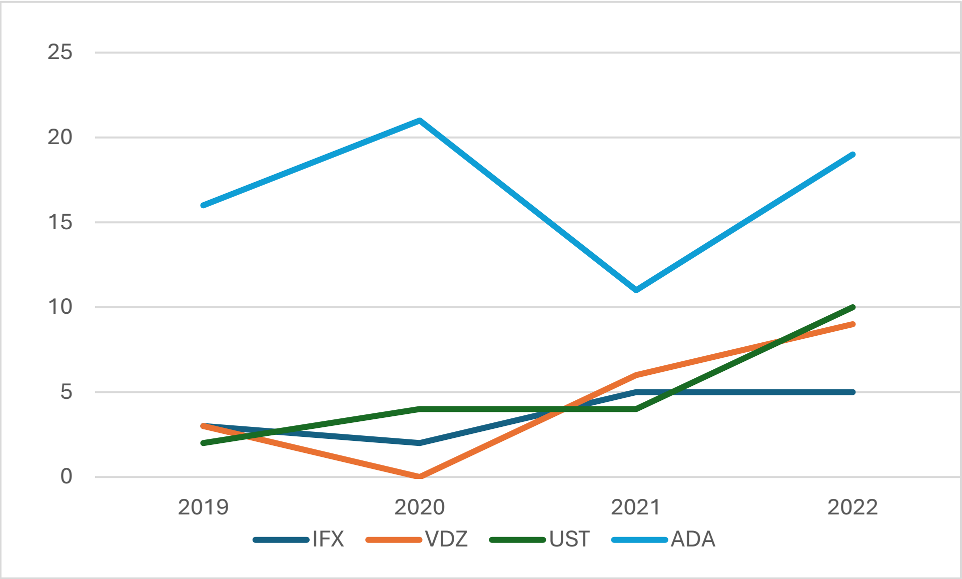

	2019	2020	2021	2022	**p-value (One Way ANOVA test)**
IFX	3	2	5	5	0.98
VDZ	3	0	6	9	0.99
UST	2	4	4	10	0.95
ADA	16	21	11	19	**0.00**

Figure S4: The trend in novel prescriptions of each biologic in bio-naïve patients with CD, through the years 2019-2022. Adalimumab is the significantly preferred medication in bio-naïve patients. There is a sharp increase of vedolizumab prescriptions, mainly in the year 2020, and a gradual upward trend in the use of ustekinumab. No significant difference in the trends of infliximab, vedolizumab and ustekinumab has been indicated.

IFX: infliximab, ADA: adalimumab, VDZ: vedolizumab, UST: ustekinumab

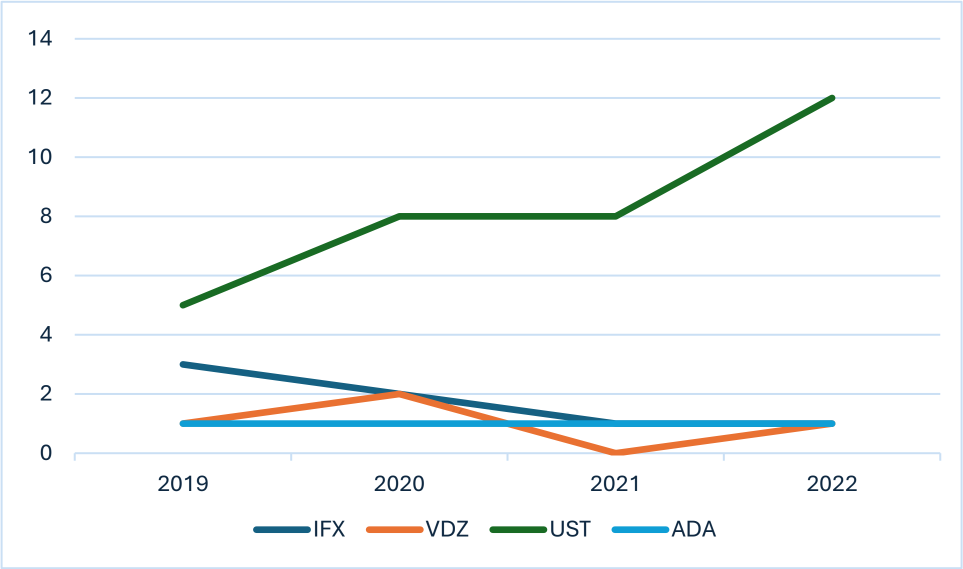

	2019	2020	2021	2022	p-value (One-Way ANOVA test)
IFX	3	2	1	1	0.90
VDZ	1	2	0	1	0.90
UST	5	8	8	12	**0.00**
ADA	1	1	1	1	0.99

Figure S5: The trend in novel prescriptions of each biologic as a second line of treatment in CD, through the years 2019-2022. Ustekinumab is significantly the most prescribed medication in bio-experienced patients. No significant difference in the prescriptions of infliximab, vedolizumab and adalimumab has been indicated.

IFX: infliximab, ADA: adalimumab, VDZ: vedolizumab, UST: ustekinumab

YEAR	NOVEL PRESCRIPTION OF BIOLOGICS IN UC FROM 2019-2022												
	IFX			VDZ			UST			ADA			
	treatment line			treatment line			treatment line			treatment line			
	1st	2nd	3rd	1st	2nd	3rd	1st	2nd	3rd	1st	2nd	3rd	Total
2019	5	3	0	14	2	2	0	0	0	1	0	1	**28**
2020	6	7	0	11	8	0	0	0	0	2	1	0	**35**
2021	6	6	1	19	1	0	0	1	1	4	1	0	**40**
2022	18	9	1	14	1	0	6	17	6	2	0	0	**74**

Table S2: Trends in the novel prescription of each biologic in both bio-naïve and bio-experienced UC patients in years 2019-2022. VDZ remains the preferred medication for bio-naïve patients, while UST is the most frequently prescribed biologic as a 2nd line treatment

IFX: infliximab, ADA: adalimumab, VDZ: vedolizumab, UST: ustekinumab

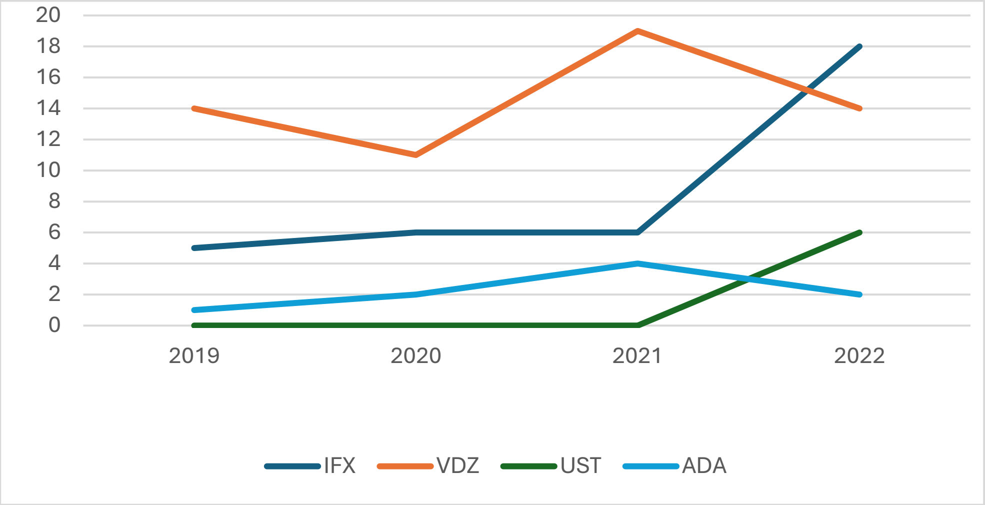

	2019	2020	2021	2022	**p-value (One-Way ANOVA test)**
IFX	5	6	6	18	0.20
VDZ	14	11	19	14	**0.002**
UST	0	0	0	6	.
ADA	1	4	4	2	0.13

Figure S6: The trend in novel prescriptions of each biologic in bio-naïve patients with UC, through the years 2019-2022. There is a sharp increase of infliximab prescriptions, particularly in 2022. Meanwhile, vedolizumab continues to be the most prescribed first-line medication, despite fluctuations in its trend.

IFX: infliximab, ADA: adalimumab, VDZ: vedolizumab, UST: ustekinumab

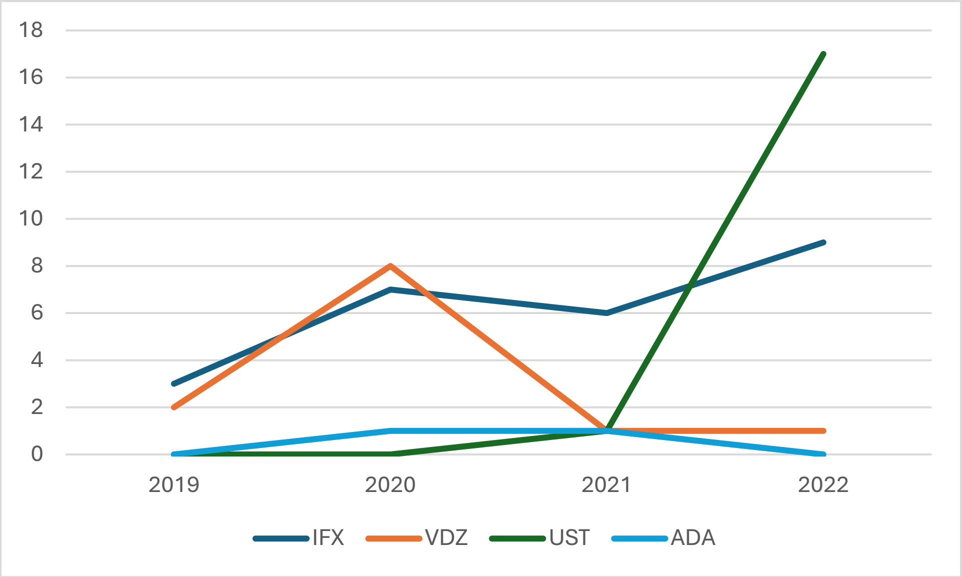

	2019	2020	2021	2022	**p-value (One Way ANOVA test)**
IFX	3	7	6	9	
VDZ	2	8	1	1	
UST	0	0	1	17	
ADA	0	1	1	0	

Figure S7: The trend in novels prescription of each biologic as a second line treatment in UC, through the years 2019-2022. No significant difference in the prescription of infliximab, vedolizumab ustekinumab and adalimumab has been indicated. Ustekinumab is numerically the most prescribed medication in bio-experienced patients, presenting a sharp increase in its trend in 2022.

IFX: infliximab, ADA: adalimumab, VDZ: vedolizumab, UST: ustekinumab

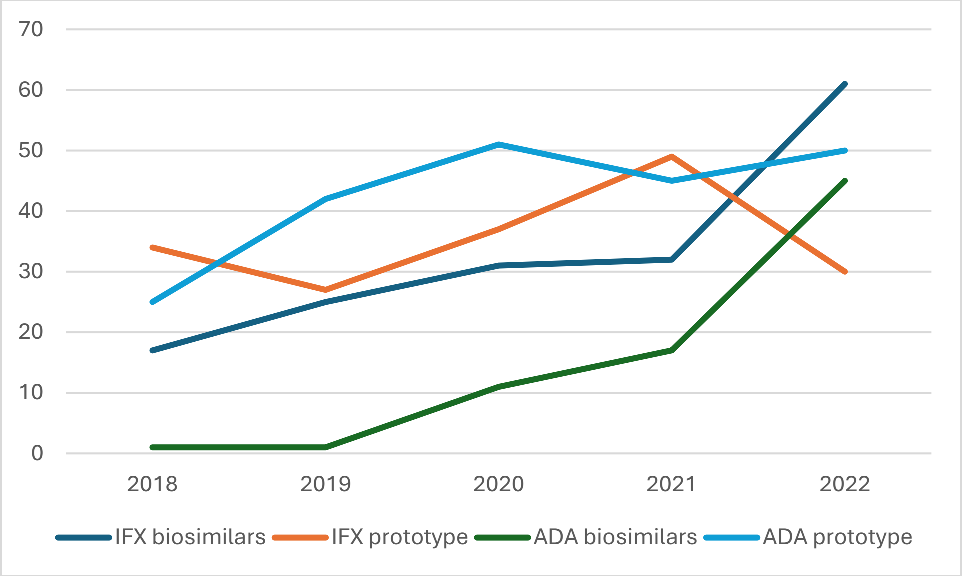

		2018	2019	2020	2021	2022
**IFX**	biosimilars	**17(33%)**	**25(48%)**	**31(45%)**	**32(39%)**	**61(67%)**
	prototype	34	27	37	49	30
**ADA**	biosimilars	**1 (4 %)**	**1 (2 %)**	**11(17%)**	**17(27%)**	**45(47%)**
	prototype	25	42	51	45	50

Figure S8 : Trends in the prescription of anti-TNF agents, both prototypes and biosimilars from 2018-2022

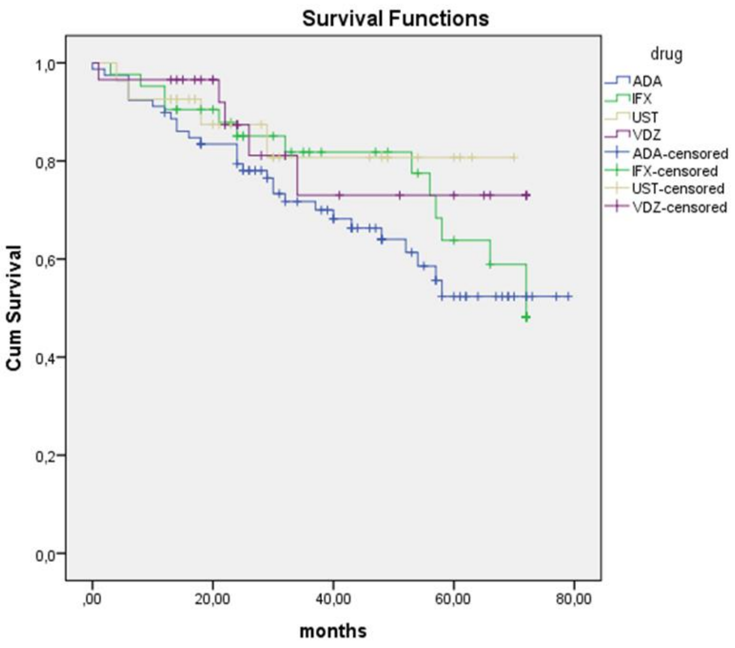

medication	Total Number of patients	Number of events (discontinuation of treatment)
ADA	79	29
IFX	42	14
UST	27	4
VDZ	29	5
overall	177	52

Overall Comparisons

	Chi-Square	df	Sig.
Log Rank (Mantel-Cox)	3,282	3	,350

Test of equality of survival distributions for the different levels of drug.

Figure S9: Kaplan–Meier curves highlight survival free of treatment discontinuation in CD patients on first line treatment, followed-up for up to 6 years. Log Rank p=0.35, showing no statistical significance

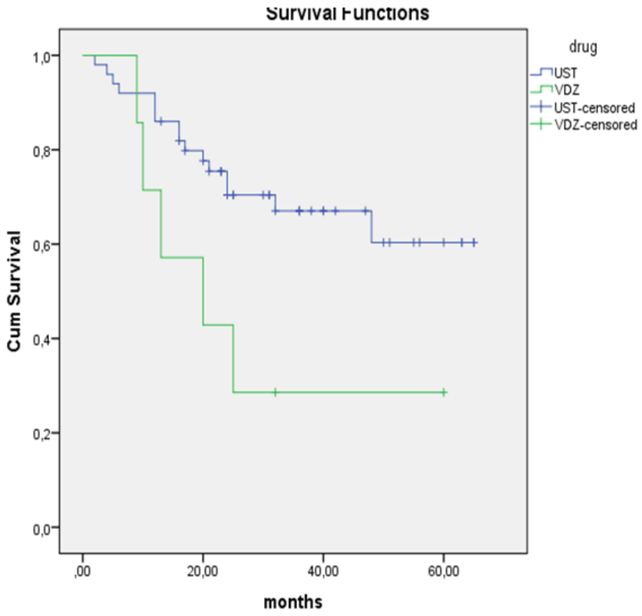

medication	Total Number of patients	Number of Events (Discontinuation of treatment)
UST	50	16
VDZ	7	5
Overall	57	21

Overall Comparisons

	Chi-Square	df	Sig.
Log Rank (Mantel-Cox)	4,251	1	,039

Test of equality of survival distributions for the different levels of drug.

Figure S10: Kaplan Meier curves demonstrate survival free of treatment discontinuation in CD patients with prior anti-TNF experience. Log Rank p=0.039 highlights the significant superiority of UST over VDZ.

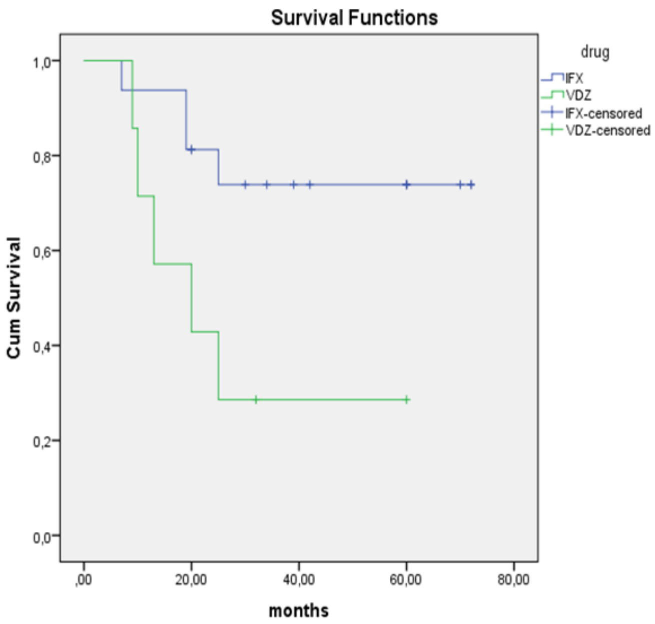

medication	Total Number of patients	Number of Events (discontinuation)
IFX	16	4
VDZ	7	5
Overall	23	9

Overall Comparisons

	Chi-Square	df	Sig.
Log Rank (Mantel-Cox)	4,677	1	,031

Test of equality of survival distributions for the different levels of drug.

Figure S11: Kaplan Meier curves demonstrate survival free of treatment discontinuation in CD patients with prior adalimumab exposure. Log Rank p=0.031 highlights the significant superiority of IFX over VDZ.

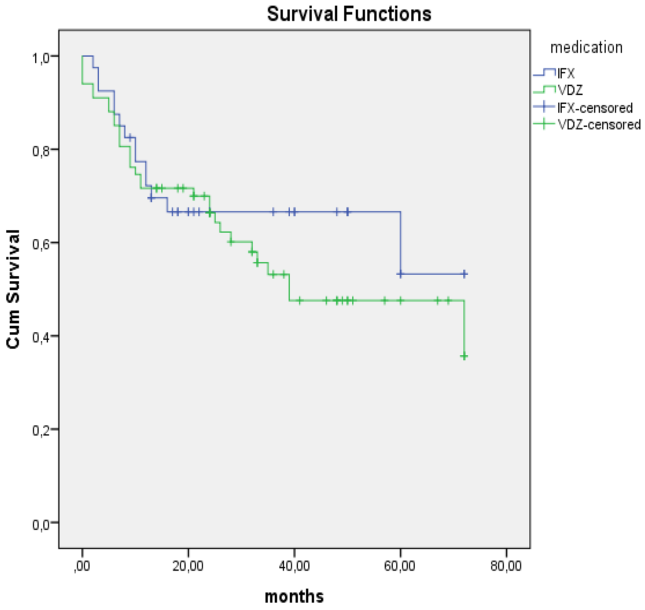

medication	Total number of patients	Number of events (discontinuation)
IFX	40	14
VDZ	67	31
Overall	107	45

Overall Comparisons

	Chi-Square	Df	Sig.
Log Rank (Mantel-Cox)	,587	1	,444

Figure S12: Kaplan Meier curves demonstrate survival free of treatment discontinuation in UC patients on IFX or VDZ as first line therapies, with follow-up of up to 6 years. Log-rank test (p = 0.444) shows no significant superiority of one medication over the other.

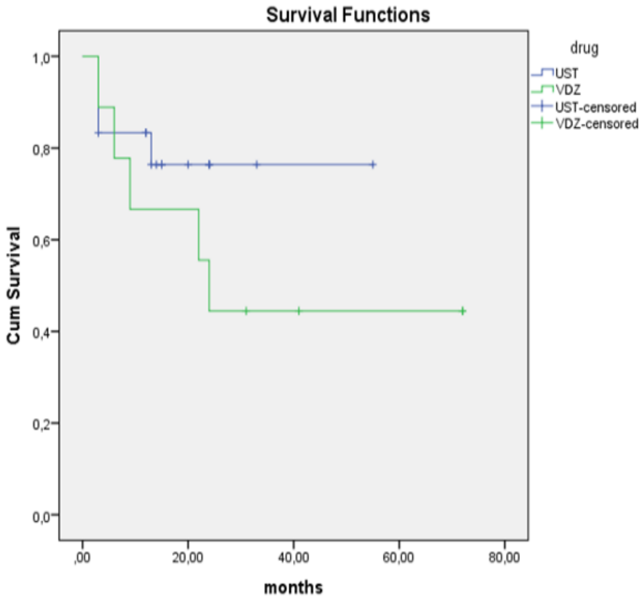

medication	Total number of patients	Number of Events (discontinuation)
UST	18	4
VDZ	9	5
Overall	27	9

Overall Comparisons

	Chi-Square	Df	Sig.
Log Rank (Mantel-Cox)	1,291	1	,256

Figure S13: Kaplan Meier curves demonstrate survival free of treatment discontinuation in UC patients with prior non-response to anti-TNF, with a follow-up for up to 6 years. Log Rank p=0.256 shows no significant superiority of one medication over the other.

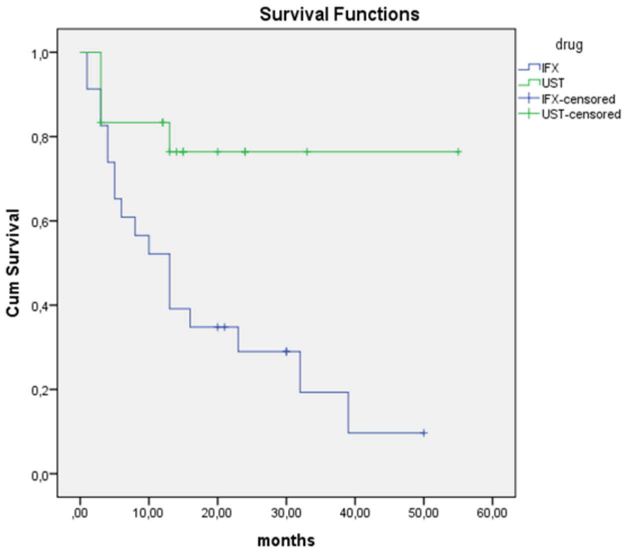

medication	Total number of patients	Number of Events (discontinuation)
IFX	24	19
UST	17	4
Overall	41	23

	Chi-Square	df	Sig.
Log Rank (Mantel-Cox)	6,539	1	,011

Figure S14: Kaplan Meier curves demonstrate survival free of treatment discontinuation in UC patients with prior non-response to vedolizumab, followed – up for up to 6 years. Log Rank p=0.011 indicates the significant superiority of UST over IFX.
